# Select Neurocognitive Impairment in HIV-Infected Women: Associations with HIV Viral Load, Hepatitis C Virus, and Depression, but Not Leukocyte Telomere Length

**DOI:** 10.1371/journal.pone.0089556

**Published:** 2014-03-04

**Authors:** Chantelle J. Giesbrecht, Allen E. Thornton, Clare Hall-Patch, Evelyn J. Maan, Hélène C. F. Côté, Deborah M. Money, Melanie Murray, Neora Pick

**Affiliations:** 1 Department of Psychology, Simon Fraser University, Burnaby, British Columbia, Canada; 2 British Columbia Mental Health and Addictions Research Institute, Vancouver, British Columbia, Canada; 3 Oak Tree Clinic, BC Women's Hospital, Vancouver, British Columbia, Canada; 4 CIHR Emerging Team in HIV Therapy and Aging (CARMA), Vancouver, British Columbia, Canada; 5 Department of Pathology and Laboratory Medicine, University of British Columbia, Vancouver, British Columbia, Canada; 6 Women's Health Research Institute, Vancouver, British Columbia, Canada; 7 Department of Obstetrics & Gynecology, University of British Columbia, Vancouver, British Columbia, Canada; 8 Division of Infectious Disease, University of British Columbia, Vancouver, British Columbia, Canada; University of North Carolina School of Medicine, United States of America

## Abstract

**Background:**

Through implementation of combination antiretroviral therapy (cART) remarkable gains have been achieved in the management of HIV infection; nonetheless, the neurocognitive consequences of infection remain a pivotal concern in the cART era. Research has often employed norm-referenced neuropsychological scores, derived from healthy populations (excluding many seronegative individuals at high risk for HIV infection), to characterize impairments in predominately male HIV-infected populations.

**Methods:**

Using matched-group methodology, we assessed 81 HIV-seropositive (HIV+) women with established neuropsychological measures validated for detection of HIV-related impairments, as well as additional detailed tests of executive function and decision-making from the Cambridge Neuropsychological Test Automated Battery (CANTAB).

**Results:**

On validated tests, the HIV+ women exhibited impairments that were limited to significantly slower information processing speed when compared with 45 HIV-seronegative (HIV−) women with very similar demographic backgrounds and illness comorbidities. Additionally, select executive impairments in shifting attention (i.e., reversal learning) and in decision-making quality were revealed in HIV+ participants. Modifiers of neurocognition in HIV-infected women included detectable HIV plasma viral load, active hepatitis C virus co-infection, and self-reported depression symptoms. In contrast, leukocyte telomere length (LTL), a marker of cellular aging, did not significantly differ between HIV+ and HIV− women, nor was LTL associated with overall neurocognition in the HIV+ group.

**Conclusions:**

The findings suggest that well-managed HIV infection may entail a more circumscribed neurocognitive deficit pattern than that reported in many norm-referenced studies, and that common comorbidities make a secondary contribution to HIV-related neurocognitive impairments.

## Introduction

Despite advances in HIV antiretroviral treatment (ART), namely the advent of combination ART (cART), HIV infection continues to be linked to deleterious functional and structural consequences for brain parenchyma [1]–[8]. Estimates indicate that as many as 50% of HIV+ individuals display some degree of neurocognitive dysfunction when impairment is derived from comparisons with normative performance standards (e.g., [9]–[11]). Nevertheless, a recent meta-analysis revealed lesser attentional, motor, and executive skill impairments in HIV+ individuals treated with cART relative to monotherapy [12]. Although impairment profiles vary amongst HIV+ individuals [13], deficits in speed of information processing [14]–[16], fine motor speed and dexterity [17], [18], aspects of learning and memory [19]–[23], and multiple domains of executive functioning [17], [24]–[30] are commonly identified. Impairment estimates appear to be attenuated when ascertained from studies in which HIV+ samples were “matched” or comparable to the control group, with respect to background demographics, lifestyle and risk variables (e.g., substance use, sexually transmitted diseases, etc.) [31], [32], or when these variables were statistically controlled [33].

A limitation of the existing literature examining neurocognitive functioning in HIV is the underrepresentation of women. Relative to men, women living with HIV are more likely to reside in marginalized settings, be less educated, and have a greater frequency of substance use including intravenous drug use (IDU) [34]–[37]. Further, compared to HIV+ men, HIV+ women are less likely to receive recommended health care. Poorer quality of care in women is associated with younger age, substance use/IDU, lower annual income, and low trust in care providers [38], [39]. These factors may directly or indirectly modify the HIV-related neurocognitive deficits observed.

In addition to describing the types and patterns of neurocognitive impairment in HIV+ populations, researchers continue to explore the associated risk factors. Several factors directly related to HIV and its treatment, such as CD4 nadir count [11], [29], [40] and detectable HIV plasma viral load [17], [41], [42], have been associated with neurocognitive impairment in some, but not all studies [10], [43]–[46]. Further, non-specific comorbid conditions may worsen neurocognitive impairment, including depression [42], [47], co-infection with hepatitis C virus (HCV) [42], [48]–[50], and substance use [51]–[53].

Further, given the aging of HIV+ cohorts, the potential for the HIV virus and/or associated factors to accelerate age-related cognitive decline has become a research imperative. Recent evidence reveals higher rates of age-associated illnesses, including cardiovascular disease, hypertension, and diabetes in HIV+ populations [54]. Whether HIV-related neurocognitive impairments are further compromised by the complex processes underlying aging has received only limited attention. Cellular aging is marked by a shortening in telomere length (TL), which has been used as a biomarker of aging. Consequently, TL in persons with HIV may reflect individual variation in cellular processes that extend beyond those typically conferred in the course of “normal” aging. Recent investigations report shorter leukocyte TL (LTL) in individuals with an HIV+ status [55]–[57], potentially implicating accelerated biological aging in HIV+ individuals. Shortened TL in immune cell subsets has been associated with HIV infection [58]–[62] and our group has reported that shorter LTL is associated with detectable viral load in HIV+ children [63].

Moreover, shortened TL reportedly increases neurocognitive impairment and risk of dementia in non-HIV populations [64]–[66] and, as such, may be a marker for atypical age-related cognitive decline [67]–[69]. One prior investigation of HIV+ women determined that shorter LTL was associated with select neurocognitive dysfunction involving learning [56]. Thus, shortened TL, beyond that expected for a given age, may predict neurocognitive functioning in persons with HIV and ultimately suggest accelerated neurocognitive aging.

The present study provides a contemporary estimate of the nature and extent of neurocognitive dysfunction in HIV+ women, relative to carefully matched HIV-seronegative (HIV−) women with similar social determinants and neurocognitive risk factors (e.g., income, HCV, etc.). Our assessment battery included measures that have been previously validated for use in HIV populations [70]. Additionally, a detailed and comprehensive examination of executive functioning was conducted to isolate core executive dysfunctions, which are generally known to be critical to real-world adaptation [71]–[73]. We also investigated potential modifiers of neurocognitive dysfunction, including HIV disease- and treatment-related factors, and comorbid conditions. Finally, we assessed LTL in our study participants, to explore any potential association with neurocognition, after controlling for relevant covariates.

## Methods

### Ethics Statement

Written informed consent was obtained from each participant. The study was approved by the Institutional Review Boards of both the University of British Columbia and Simon Fraser University (ethics reference numbers: H09-02867 & 2010s0464, respectively), and was conducted in accordance with the Helsinki Declaration (59^th^ WMA General Assembly, Seoul, 2008).

### Participants

One hundred and twenty-six (126) women were administered neurocognitive batteries between August 2010 and May 2012. All participants had adequate English language fluency for the purpose of valid psychometric testing. Exclusion criteria included; age <30 years, head injury with loss of consciousness >10 minutes, being currently pregnant, serious mental illness (e.g., bipolar disorder or schizophrenia), or known central nervous system pathology, including progressive multifocal leukoencephalopathy, cancer affecting the brain, neurosyphilis, active cytomegalovirus infection, multiple sclerosis, stroke, and seizures/epilepsy. Eighty-one (81) HIV+ women were recruited from the Oak Tree Clinic in Vancouver, British Columbia (BC), a provincial multidisciplinary HIV clinic primarily for women and children. Forty-five (45) HIV− control women were recruited throughout the Lower Mainland of BC via advertisements strategically posted in various venues, including women's health clinics and Craigslist. Control participants were tested for HIV antibody upon recruitment in the study unless they had a negative test in the past 3 months with no other illness during this time period. All neurocognitive testing was conducted at the BC Women's Hospital and Health Centre. Participants received a modest honorarium for their participation.

### Material and Procedures

#### Medical, clinical, and substance use evaluations

General medical history was obtained via self-report for all participants. For HIV+ participants, HIV disease duration (estimated from date of HIV diagnosis) and ART history were retrieved from medical chart and clinic pharmacy records. Both HIV plasma viral load (copies/mL) and CD4 count (cells/µL) were obtained from blood work collected within 5 months of neurocognitive testing. HCV seropositivity (HCV+) was self-reported by control participants and retrieved from medical chart for HIV+ participants. For participants identified as being HCV seropositive, a follow-up qualitative PCR (qPCR) on plasma collected on study visit day was performed at the BC Centre for Disease Control to ascertain whether HCV infection was active.

Substance use history was obtained from all participants with a self-report questionnaire. To summarize substance use information five different ‘Substance Use’ variables were developed for the different substance use histories assessed (i.e., amphetamines, cocaine, crack, heroin, and marijuana). For every participant each substance was dichotomized into lifetime minimal use (monthly or rarely/never) or lifetime severe use (daily or weekly). A ‘Lifetime Substance Use’ variable was created, and used to represent substance use in subsequent statistical analyses, by dichotomizing participants into those who never used any substance and those who reported use of at least one substance in their lifetime. Recency of last substance use was also determined using a ‘Day of Evaluation’ questionnaire, in which participants self-reported whether they used various substances within 24 hours of cognitive testing.

To assess LTL, DNA was extracted from peripheral blood collected on the same day as neurocognitive testing. Relative mean LTL was measured by monoplex qPCR as described previously [74].

#### Psychometric assessments

English language fluency was assessed with a brief self-report questionnaire of subjective language preferences for various activities (e.g., reading, speaking, etc.). The Center for Epidemiologic Studies Depression Scale (CES-D) [75] was used to evaluate current levels of depression symptoms. To determine group comparability on premorbid intellectual abilities, the Wechsler Test of Adult Reading (WTAR) [76] was administered. For the vast majority of participants, the WTAR Word Reading plus demographic-predicted Full Scale IQ (FSIQ) was used as the premorbid estimate. For five HIV+ participants and one HIV− participant for whom English was a second language, but who were nonetheless fluent, the WTAR demographic-only predicted FSIQ was used.

#### Neurocognitive assessment

All participants completed a comprehensive neuropsychological battery. Test formats fell into two categories: conventional paper and pencil tests validated in the existent literature [70] and the computerized Cambridge Neuropsychological Test Automated Battery (CANTAB) [77]. Using the conventional tests, we evaluated fine motor speed and dexterity (Grooved Pegboard Test) [78], learning and memory (Hopkins Verbal Learning Test-Revised; HVLT-R) [79], speed of information processing (Symbol Search and Coding; WAIS-IV) [80], and working memory (Letter-Number Sequencing; WAIS-IV) [80]. Along with the HVLT-R, learning and memory were further examined using the Paired Associates Learning (PAL) subtest from the CANTAB, a computerized visual memory test that evaluates conditional learning of pattern-location associations. The number of patterns recalled after a single viewing and the total number of errors made were assessed.

Additionally, the CANTAB was used in conjunction with Letter-Number Sequencing to comprehensively evaluate executive abilities (Table 1). Several “core” (e.g., attention, working memory, information updating and monitoring, response inhibition, attentional set-shifting) and “compiled” (e.g., decision-making, planning) domains of executive functioning were targeted. This battery of tests captures the organization taxonomy and breadth of executive functions [81]–[83].

**Table 1:** Executive functioning test descriptions.

	Description of Test	Cognitive Scores
**Attention** (Rapid Visual Information Processing; RVP)	Sustained attention test where digits (2–9) are pseudo-randomly displayed in middle of the screen. Push button when one of three target sequences (e.g., 2-4-6) is detected.	**A′:** signal detection measure of sensitivity; **B″:** strength of trace needed to elicit response.
**Working Memory** (Letter-Number Sequencing; LNS)	Read letter – number sequences. Repeat numbers in order from lowest to highest, followed by the letters in alphabetical order.	Number of correctly recalled sequences.
**Information Updating and Monitoring** (Spatial Working Memory; SWM)	Randomly arranged coloured boxes are displayed on the screen. Through an elimination process, must locate a hidden token in each of a number of boxes in order to fill a column on the right side of the screen. Number of boxes displayed gradually increases to 8. Tokens are never hidden under the same box twice within a search stage.	**Strategy:** following a pre-determined sequence to locate all hidden tokens; **Total Between Errors:** number of times a box is returned to where a token was previously found.
**Response Inhibition** (Stop Signal Task; SST)	Indicate whether an arrow is pointing left or right using press pad; if an auditory signal (beep) sounds must inhibit the dominant response.	**Inhibition Speed:** inhibition of response that has been initiated.
**Attentional Set-Shifting** (Intra-Extra Dimensional Set Shift; IED)	Task of rule acquisition and reversal involving 9 stages in which a new rule must be learned regarding 2 dimensions (lines and shapes). Learning at each stage occurs by satisfying a set criterion (6 consecutive correct responses) based on either discriminating dimensions, reversal of correct dimension based on feedback, or shifting between dimensions. *(Note: Failure at a particular stage ended the task with no opportunity to complete subsequent stages. Therefore, for the relevant stages adjusted error scores for all non-completers were established by adding one error to the error total observed in the participant who made the most errors yet completed the given stage).*	**EDS Errors:** errors made on Stage 8 (extradimensional shift). Failure to shift attention to a novel exemplar of a previously unrewarded dimension; **Pre-EDS Errors:** total errors made before Stage 8. Failure to select stimuli that are compatible with the current, intradimensional, rule (e.g. lines); **Discrimination Errors:** sum of errors on Stages 1, 3, 4. Failure to acquire a rule based on discriminating shapes; **Reversal Errors:** sum of errors on Stages 2, 5, 7. Failure to shift to previously irrelevant stimuli.
**Decision-Making** (Cambridge Gambling Task; CGT)	Decide if a token is hidden under a red or blue box, and bet a certain proportion of available points that the choice is correct.	**Quality of Decision-Making:** proportion of times chose more likely outcome; **Risk Adjustment:** adjust risk taken based on probability of winning (i.e., the degree to which risk differs across different ratios of red and blue boxes). Higher scores represent proportion of times bet larger amount of available points on high ratio trials (e.g., 9∶1) whereas lower scores represent betting large proportion of points on low ratio trials (e.g., 6∶4).
**Planning** (Stockings of Cambridge; SOC)	A spatial planning test that involves arranging 3 coloured balls in the bottom display to look like the top display in a pre-determined minimum number of moves.	**Optimal Solution:** number of trials successfully completed in minimum number of moves.

To quantify overall level of neurocognition a standardized composite z-score index (Composite Cognitive Index; CCI) was calculated. Briefly, z-scores were computed for 13 non-redundant neurocognitive outcome measures, among all the tests administered, on the basis of the mean and standard deviation of the HIV− group [84], [85], using transformed data when required. For each HIV+ and HIV− participant the mean of their individual z-scores was generated and used as a composite (overall) index of neurocognitive performance. This in turn was employed to ascertain overall group differences in neurocognition as well as in subsequent analyses of potential modifiers of neurocognition.

### Statistical Analyses

#### Group Comparisons

All comparisons made between HIV+ and HIV− groups were conducted on raw neurocognitive data and on the CCI. For normally distributed data, independent *t*-tests were employed. For a subset of CANTAB outcome measures, mathematical transformations (e.g., square root and logarithm10) failed to adequately normalize the distributions; therefore, Mann-Whitney *U* nonparametric tests were conducted on the raw, untransformed scores. Effect sizes (ES) for group comparisons were also calculated for each neurocognitive outcome measure. Cohen's *d* was employed for normally distributed data (corresponding to ES of small = .2, medium = .5, large = .8) [86]. For non-normal distributions, comparable ES were calculated [87] based upon the proportion of the HIV− group that fell below (i.e., worse performance) the median for the HIV+ group (γ^c^ < Median (γ^e^); where c = HIV− group and e = HIV+ group). Despite the fact that the distributions for these select scores were non-normal, the proportions were then converted into z-values appropriate for abnormal distributions, which estimates the median performance of the HIV+ group relative to the control distribution (e.g., z-value of −1.0 represents the 16^th^ percentile of the control group distribution) [87].

#### Normative-Referenced Scores

For conventional neurocognitive measures (i.e., Grooved Pegboard, HVLT-R, Symbol Search, Coding, LNS) performance of the HIV+ group was further inspected in the context of published normative data [78]–[80] using the following procedures. First, each HIV+ participant's raw score was converted to its corresponding age-corrected standard score based upon the published norm-referenced data. Next, for each measure, the arithmetic mean for the HIV+ group was computed, which represents the HIV+ group's performance on each measure relative to the published normative sample. Lastly, because scores are given in various standardized units (e.g., t-scores, scaled scores, etc.), the HIV+ group means for each measure were converted to a common z-score metric (mean = 0 and standard deviation = 1.0 with comparable percentiles to Cohen's *d*). These normative-referenced z-scores represent the deviation of the HIV+ group from that of the published normative standardization sample on the conventional neurocognitive measures.

#### Neurocognitive Correlates

In the HIV+ group partial correlations (including point biserial partial correlations) [88] controlling for age and education were used to explore the associations between discrete aspects of neurocognition, as indexed by raw scores, and the following: 1) *disease- and treatment-related factors* (CD4 nadir cell count, current CD4 cell count, current detectable HIV plasma viral load, duration since HIV diagnosis, years on ART), 2) *comorbid conditions* (depression, active HCV infection, lifetime substance use), and 3) *LTL*. Exceptions included employing Spearman's *rho* for scores from the Intra-Extra Dimensional Set Shift (IED) test (after confirming no correlation with age and education) as transformations failed to adequately normalize the distributions. Additionally, prior to running analysis, several data modifications were employed to ensure normal distributions. For Grooved Pegboard, time taken with dominant and non-dominant hand was combined for a total time score. For RVP (A′) transformed data (cubed) was used. For the decision-making score, Quality of Decision-Making and Risk Adjustment scores from the Cambridge Gambling Task were combined (raw scores were z-score transformed and summed).

#### LTL Investigations

An analysis of covariance (ANCOVA) was used to compare LTL differences between HIV+ and HIV− groups. Age was used as a covariate in the model, given that it was marginally associated with LTL in the entire sample (*r* = −.16; *p* = .07).

#### Prediction of Overall Cognition

Finally, two hierarchical linear regression analyses (HLRA) were conducted separately in the HIV+ and HIV− samples to investigate the contribution of demographic factors, disease- and treatment-related factors, comorbid conditions, and LTL to overall neurocognition as measured by the CCI. A separate analysis for each sample was conducted given that HIV disease- and treatment-related factors could only be investigated in the HIV+ group, and the apparent differential relationships observed for each sample in the associations between clinical and demographic variables and CCI. Based upon zero-order correlations potential independent variables were pre-screened prior to inclusion into the model and only those that shared at least 5% of their variance with CCI were entered. For both samples the pre-screened variables considered were education, depression, active HCV infection, and lifetime substance use. In the HIV+ sample we also pre-screened CD4 nadir cell count, current CD4 cell count, detectable HIV viral load, duration since HIV diagnosis, and years on ART. Age and LTL were included in both analyses to evaluate the extent to which LTL (entered in the final block) contributed to overall neurocognition beyond that of age and the pre-screened variables. All statistical analyses were conducted with IBM SPSS Statistics V. 20.0 (IBM Corporation, Armonk, NY).

## Results

### Group Comparability

HIV+ and HIV− participants were well matched on age, premorbid intellectual functioning (WTAR FSIQ), education, ethnicity, and income, and were comparable on several comorbid conditions that are potential modifiers of impairment, including depressive symptoms, HCV serostatus, and lifetime substance use (see Table 2). The only exception was a significantly higher frequency (Fisher's exact test: *p* = .01) of self-reported recent cocaine use in the HIV− group (n = 7/45) compared to the HIV+ group (n = 2/81). However, given that the half-life of cocaine is approximately one hour [89], [90], and that the mean reported time since recent use in the entire group was approximately 12 h (M = 12.69 h; SD = 3.34 h) this was not likely to confound the results (see review [91]). Descriptive statistics for disease- and treatment-related variables in the HIV+ group are also shown in Table 2.

**Table 2:** Demographic and clinical characteristics of HIV+ and HIV− participants.

	HIV+ (n = 81)	HIV− (n = 45)	*p-value*
**Age (years)** a	44.4 (7.9)	46.0 (10.0)	.31
**Age Range (years)**	30–66	31–67	
**WTAR (FSIQ)** a	98.9 (10.3)	102.1 (11.1)	.11
**Education (years)** a	12.5 (2.3)	13.0 (3.0)	.34
**Education Range (years)**	7–18	8–18	
**Race/Ethnicity (%)** b **^, ^** †			.07
White (%)	51.9	55.6	
Aboriginal (%)	29.6	40.0	
Black (%)	13.6	4.4	
Asian (%)	4.9	0.0	
**Household Income (% ≤$15, 000)** b **^, ^** †	45.6	62.0	.08
**Mild Head Injury (%)** b **^, ^** †	20.8	36.4	.06
**CES-D** a	15.2 (12.2)	11.7 (10.9)	.10
**HCV (% ever/% active at testing)** b	34.6/24.7	24.4/15.9	.24/.25
**Substance Use**			
Lifetime Smoker (%)^b^	70.4	79.5	.27
Alcohol (% Currently Using)^b^	58.0	56.8	.90
Amphetamine (% Heavy Use)^b^	1.3	6.8	.10
Cocaine (% Heavy Use)^b^	30.8	31.8	.90
Crack (% Heavy Use)^b^	26.9	34.1	.40
Heroin (% Heavy Use)^b^	15.4	13.6	.79
Marijuana (% Heavy Use)^b^	41.0	52.3	.23
Lifetime Substance Use (% Heavy Use)^b^	54.3	64.4	.27
Alcohol (% Recent use)^b^	16.0	22.2	.39
Amphetamine (% Recent use)	0.0	0.0	n/a
Cocaine/Crack (% Recent use)^c^^, ^ *	2.5	15.6	.01
Heroin (% Recent use)^c^	3.7	0.0	.55
Marijuana (% Recent use)^b^	22.2	13.3	.22
**Duration since Diagnosis (years)**	11.8 (5.5)		
**Duration of ART (years)**	7.3 (5.6)		
**CD4 nadir (cells/µL)**	185.2 (136.3)		
≥500 (%)	2.5		
200–499 (%)	40.7		
≤199 (%)	56.8		
**Current CD4 (cells/µL)**	516.2 (243.0)		
≥500 (%)	48.1		
200–499 (%)	45.7		
≤199 (%)	6.2		
**% Undetectable HIV RNA**	76.5		
**% cART-treated**	87.7		
**Leukocyte Telomere Length** d	2.9 (0.62)	2.9 (0.48)	.62

*Note.* Values represent Mean (standard deviation) unless otherwise indicated; WTAR = Wechsler Test of Adult Reading (full-scale IQ); Asian includes South Asian; CES-D = Center for Epidemiologic Studies Depression Scale (unadjusted means are displayed, but square root transformed scores were used for statistical analysis); HCV = Hepatitis C Virus; % Heavy Use = Daily or weekly use of substance over lifetime; % Recent use = self-reported substance use within 24 hours of neurocognitive testing; Undetectable HIV RNA = viral load ≤200 copies/mL; Groups compared using:

a independent *t*-tests,

b χ2 Pearson chi-square;

c Fisher's exact test (2-sided);

d ANCOVA; n/a = not applicable;

† *p*<.10;

*p<.05.

### HIV+ and HIV− Differences in Discrete Aspects of Neurocognition

Table 3 reveals select neuropsychological differences between the HIV+ and HIV− participants. Specifically, HIV+ women exhibited slower visual scanning/discrimination speed (e.g., Symbol Search; *t*_(123)_ = −2.21, *p* = 0.03), which was the only conventional test detecting impairment. On the CANTAB measures, moderate to large impairments in select executive skills were revealed. Specifically, on the IED task, HIV+ participants made significantly more errors on the pre-extradimensional shift stages (*U* = 1277.50, *Z* = −2.62, *p* = 0.009), which reflected greater reversal errors (*U* = 1274.50, *Z* = −2.72, *p* = 0.007), and a trend towards greater discrimination errors (*U* = 1436.00, *Z* = −1.79, *p* = 0.072). Note that these analyses included data from subsets of participants for whom adjustments were made to error scores for the stages that were not completed (see Table 1 for score adjustment details, also see [92] for similar adjustments). Nonetheless, the results were maintained for participants who completed all relevant IED stages: pre-extradimensional shift stage errors (*U* = 982.00, *Z* = −2.08, *p* = .037), reversal errors (*U* = 996.50, *Z* = −2.10, *p* = .035), and discrimination errors (*U* = 1280.50, *Z* = −1.68, *p* = .093). Reversal stage errors suggested a core deficit in attentional set-shifting; specifically, the HIV+ group suffered degradation in shifting to previously irrelevant stimuli within the context of a rule change. Additionally, the HIV+ group demonstrated significantly poorer quality of decision-making on the Cambridge Gambling Task (CGT; *U* = 1379.50, *Z = *−2.09, *p* = 0.04). That is, they more often gambled on the less likely outcome. There were no significant group differences in fine motor speed and dexterity, learning and memory, or the remaining sub-domains of executive function. Lastly, the groups did not significantly differ on the CCI (see Table 3 notation for measure that constitute the CCI).

**Table 3:** Comparison of neurocognitive performance between HIV+ and HIV- groups.

		HIV+				HIV−				Effect Sizes	
Test	Measure	Mean	Median	SD	N	Mean	Median	SD	N	HIV+ vs. HIV−	HIV+ vs. Norms^c^
**Fine Motor Dexterity and Speed**											
GP	Dominant (sec)∧	68.62	69.00	12.17	81	67.41	65.50	13.15	44	−0.10^a^	−0.81
	Non-Dominant (sec)	74.51	70.00	13.85	79	71.60	70.00	11.35	40	−0.23^a^	−0.48
**Learning and Memory**											
HVLT	Total Recall∧	23.46	24.00	5.60	81	25.24	26.00	5.11	45	−0.33^a^^, ^ †	−1.04
	Delayed Recall	8.25	8.00	2.47	81	9.09	9.00	2.29	45	−0.35^a^^, ^ †	−0.91
PAL	First Trial∧	12.75	13.00	3.58	81	12.58	12.00	3.88	45	0.05^a^	
	Total Error	25.90	17.00	23.60	81	24.33	17.00	21.44	45	−0.08^b^	
**Speed of Information Processing**											
SS	Raw Score∧	28.98	28.00	8.40	80	32.56	31.00	9.23	45	−0.41^a^^, ^ *	−0.42
Coding	Raw Score∧	64.06	62.00	14.80	81	65.93	63.00	14.42	45	−0.13^a^	−0.33
**Attention**											
RVP	A′∧	0.89	0.90	0.06	80	0.89	0.90	0.05	45	−0.03^b^	
	B″	0.88	0.94	0.18	79	0.93	0.96	0.07	45	−0.31^b^	
**Working Memory**											
LNS	Raw Score∧	17.75	17.00	3.40	81	17.64	18.00	2.91	45	0.03^a^	−0.72
**Information Updating and Monitoring**											
SWM	Strategy	33.70	34.00	6.17	81	35.04	36.00	5.40	45	0.23^a^	
	Between Errors∧	32.09	29.00	21.33	81	32.09	32.00	18.68	45	0.00^a^	
**Response Inhibition**											
SST	Inhibition Speed∧	204.91	196.12	55.46	78	195.01	186.78	51.39	45	−0.19^a^	
**Attentional Set-Shifting**											
IED	EDS errors	15.51	11.00	14.37	79	16.02	13.00	13.06	45	0.08^b^	
	Pre-EDS errors	28.84	7.00	41.68	79	16.98	5.00	32.90	45	−0.57^b^^, ^ **	
	Reversal errors∧	16.81	5.00	23.32	79	9.36	3.00	16.90	45	−1.01^b^^, ^ **	
	Discrimination errors∧	10.49	2.00	18.11	79	6.76	1.00	15.29	45	−0.43^b^^, ^ †	
**Decision-Making**											
CGT	Quality∧	0.87	0.94	0.15	79	0.92	0.97	0.12	45	−0.57^b^^, ^ *	
	Risk Adjustment	0.76	0.52	0.99	79	0.95	0.85	0.91	45	−0.21^a^	
**Planning**											
SOC	Optimal Solutions∧	8.52	9.00	1.90	79	8.71	9.00	2.21	45	−0.09^a^	
**Composite Cognitive Index (CCI)**		−0.27	−0.01	1.08	81	0.00	0.12	1.00	45	0.25^a^	

*Note*. All cognitive scores represent raw data; GP = Grooved Pegboard; HVLT = Hopkin's Verbal Learning Test- Revised; PAL = Paired Associates Learning; SS = Symbol Search; RVP = Rapid Visual Information Processing; LNS = Letter-Number Sequencing; SWM = Spatial Working Memory; SST = Stop Signal Task; IED = Intra-Extra Dimensional Set Shift; CGT = Cambridge Gambling Task; SOC = Stockings of Cambridge; sec = seconds; EDS = Extradimensional Shift; Inhibition Speed (milliseconds); SD = Standard deviation;

∧ = inclusion in CCI;

a Cohen's *d*;

b non-parametric effect size (z-value);

c *z*-score (mean = 0; SD = 1) based on normative data;

† *p*<.10;

*p<.05,

**p<.01 (based on independent *t*-tests or Mann-Whitney *U* nonparametric tests when appropriate).

### HIV+ Participants' Neurocognition Relative to Normative-Referenced Standards

When the HIV+ group's neurocognitive performances were indexed to published normative-referenced standards, the magnitude of impairments appeared much greater than when deficits were estimated on the basis of comparisons to a well-matched control group (see Table 3). This pattern emerged on tests of fine motor speed and dexterity (Grooved Pegboard), learning and memory (HVLT-R), information processing speed (Coding), and working memory (LNS). The only exception was for the task involving visual scanning/discrimination speed (Symbol Search) where the magnitude of impairment was comparable when either the matched control group (Cohen's *d* = −0.41) or published normative data (*z*-score = −0.42) were employed.

### Associates of Discrete Aspects of Neurocognition in HIV+ Participants

The relationship between disease- and treatment-related variables and neurocognition in the HIV+ group are shown in Table 4. Lower current CD4 count was significantly associated with more errors on reversal learning, but none of the other neurocognitive abilities. Detectable, or non-suppressed, HIV viral load (>200 copies/mL) was related to poorer neurocognitive functioning. This result was observed in overall neurocognition (CCI) and among specific domains including information processing speed, learning, working memory, and information updating and monitoring. Further analysis comparing the virally suppressed group (i.e., viral load ≤200 copies/mL; n = 62) to the detectable viral load group (n = 19) revealed a significant difference in overall neurocognitive functioning (CCI; *t*_(79)_ = 3.0, *p* = .004), with the detectable virus group performing significantly worse (M = −0.89, SD = 0.97) relative to the virally suppressed group (M = −0.08, SD = 1.04). A longer duration since HIV diagnosis was associated with better learning and memory, a larger working memory capacity, and selectively faster information processing speed (e.g., Coding). The lifetime number of years treated with ART, which was confounded with duration since HIV diagnosis (*r* = .805, *p*<.001), was similarly related to better learning, and selectively faster information processing speed.

**Table 4:** Correlations between neurocognitive performance and disease- and treatment-related variables, and comorbid conditions in the HIV+ group.

Test (score)	Nadir CD4^a^	Current CD4	Detectable Viral Load	Years since Diagnosis	Years on ART	Depression^a^	Active HCV	Lifetime Substance Use	LTL
**Fine Motor Dexterity and Speed**									
Pegboard	−.14	−.16	.21†	−.21†	−.17	.10	.30*	.14	−.07
**Learning and Memory**									
HVLT (total recall)	.01	.00	−.27*	.28*	.23*	−.12	−.07	.11	.09
PAL (first trial)	.09	−.05	−.31**	.24*	.20†	−.04	−.17	.05	.18
**Speed of Information Processing**									
SS	.10	.08	−.07	.12	.08	−.13	−.29*	.17	−.03
Coding	.00	.15	−.25*	.34**	.29*	−.06	−.33**	−.00	.03
**Attention**									
RVP (A′)^b^	.09	.01	−.18	.03	.02	−.20†	−.28*	−.02	.09
**Working Memory**									
LNS	−.13	.10	−.36**	.26*	.23†	−.02	−.28*	.12	.00
**Information Updating and Monitoring**									
SWM (between err)	.09	.01	.25*	−.13	−.01	.30*	.07	−.01	.02
**Response Inhibition**									
SST (inhibition speed)	−.16	−.06	−.12	.04	.09	−.05	.23†	.25*	−.07
**Attentional Set-Shifting**									
IED (reversal err)^c^	−.03	−.24*	.07	.12	.08	.15	.13	.10	−.11
IED (discrimination err)^c^	−.04	−.12	.07	.10	.03	.23*	.16	.06	−.16
**Decision-Making**									
CGT (quality + risk adj.)	−.08	−.06	−.08	.06	.07	−.22†	−.09	−.02	.08
**Planning**									
SOC (optimal solutions)	.02	.09	−.19	.22†	.05	−.30*	−.24*	−.03	.03
**CCI**	.07	.21†	−.28*	.17	.16	−.23*	−.30**	−.01	.12

*Note.* Values represent partial *r*'s controlling for age and education (unless indicated otherwise); HVLT = Hopkin's Verbal Learning Test- Revised; PAL = Paired Associates Learning; SS = Symbol Search; RVP = Rapid Visual Information Processing; LNS = Letter-Number Sequencing; SWM = Spatial Working Memory; SST = Stop Signal Task; IED = Intra-Extra Dimensional Set Shift; CGT = Cambridge Gambling Task; SOC = Stockings of Cambridge; CCI = Composite Cognitive Index; err = errors; Detectable viral load = >200 copies/mL; ART = Antiretroviral Therapy; HCV = Hepatitis C virus;

a Square root transformed data;

b Cubed transformation;

c Spearman's *rho*;

† *p*<.10;

*p<.05;

**p<.01.

The relationship between comorbid conditions and neurocognition in the HIV+ group are also reported in Table 4. Greater depressive symptoms were significantly associated with worse neurocognitive functioning overall (CCI) and among specific domains; including poorer information updating and monitoring, worse discrimination of stimuli, and lesser planning capacity. Additionally, active HCV infection was associated with poorer overall neurocognition (CCI) and specifically with slowing of motor and information processing speed, poorer attention, and reduced working memory capacity and planning behaviour. Also, heavy lifetime substance use was selectively associated with decreased response inhibition. Finally, no significant partial correlations were identified between discrete aspects of neurocognition and LTL in the HIV+ group (see Table 4).

### HIV+ and HIV− Differences in LTL

Prior to conducting the ANCOVA we identified one LTL value in the HIV+ group that was a significant outlier and adjusted it accordingly [93]. Further, two HIV+ cases and one HIV− case had missing LTL values and were excluded from this analysis and subsequent analyses involving LTL. The ANCOVA comparing HIV+ and HIV− groups (see Table 2) revealed no significant group differences in LTL (*F*_ (1, 120)_ = 0.25, *p* = .62), after accounting for age (*F*_ (1, 120)_ = 2.57, *p* = .11).

### Modifiers of Overall Neurocognition in HIV+ Participants

The following HLRA results were based upon entering four blocks of variables in the prediction of overall neurocognition of HIV+ participants, after pre-screening (i.e., those sharing at least 5% of variance with CCI) for possible predictors. In the first block, demographic variables were entered, followed by variables capturing comorbid conditions, subsequently HIV disease- and treatment-related variables were entered, and finally LTL was entered. On the first block, education and age together explained 20.6% of the variance in CCI (*F*_(2, 76)_ = 9.86, *p*<.001). On block two, the comorbid factors of depression and active HCV infection explained an additional 11.4% of the variance (*F*_(2, 74)_ = 6.19; p = .003). Subsequently, detectable HIV viral load, entered on block three, explained a further 7.0% of the variance (*F*_(1, 73)_ = 8.34, *p* = .005). On the final block LTL was entered, but it did not account for a significant amount of additional variance in CCI (*F*_(1, 72)_ = 0.19, *p* = .67). The final model was significant (*F*_(6, 72)_ = 7.71, *p*<.001), accounting for 39.1% (34% adjusted) of the variance in CCI. In addition to LTL (β = .04, *p* = .67), the full model indicated that age (β = −.13, *p* = .19) was not a significant predictor of CCI, but education (β = .34, *p* = .001), depression (β = −.21, *p* = .026), HCV infection (β = −.28, *p* = .004), and detectable HIV viral load (β = −.26 *p* = .009) were significant.

### Modifiers of Overall Neurocognition in HIV− Participants

For completeness, a similar HLRA approach was adopted in the investigation of overall neurocognition in HIV− participants. Pre-screened variables sharing at least 5% variance with CCI included education and lifetime substance use. Age and LTL were also included in the model. Consequently, the HLRA was constructed by entering three variable blocks in the following order: 1) age and education, 2) lifetime substance use, and 3) LTL. Age and education explained 51.7% of the variance in CCI (*F*_(2, 41)_ = 21.91; p<.001). The addition of lifetime substance use contributed minimally (<1%) to the variability of CCI (*F*_(1, 40)_ = .32; p* = *.58). Lastly, LTL explained an additional 5.0% of the variance in overall neurocognition (*F*_(1, 39)_ = 4.51; *p* = .04). The full model was significant (*F*_(4, 39)_ = 12.93, *p*<.001), explaining 57% (53% adjusted) of the variance in overall neurocognition (CCI). The model indicated that lifetime substance use (β = .14, *p* = .31) was not a significant predictor of CCI, but age (β = −.23, *p* = .04), education (β = .69, *p*<.001), and LTL (β = .23 *p* = .04) were significant.

## Discussion

Compared to existent research, the current findings are fairly unique in revealing relatively select neurocognitive impairments in HIV+ women who were well matched to controls on age, premorbid intellectual abilities, education, ethnicity, and household income, and on other potential confounds (e.g., depressive symptoms, active HCV infection, and lifetime substance use). The current study revealed impairments that were limited to slowing of information processing and deficits in executive function. Decreased speed of information processing is a well-established finding within HIV+ populations [14]–[16]; however; in contrast to most prior reports, this was the only significant deficit detected on conventional tests. Nonetheless, by deploying computerized (CANTAB) measures fairly novel to the HIV literature (see [18], [94] for additional use of select subtests and scores in HIV+ samples), the findings extended our understanding of core [82], [95] HIV-related executive impairments in women.

Functionally, HIV+ women had difficulty responding to previously irrelevant stimuli (IED Stages 2, 5, 7) despite feedback suggesting that the stimulus was now relevant to success. This executive impairment pattern, indicative of a reversal learning deficit, has been associated with activations of the ventral prefrontal cortex [96]–[99]. In contrast, HIV+ women showed only marginal trends towards impairment in rule acquisition (IED discrimination stages). Further, they were relatively successful in shifting attention to a novel exemplar of a previously unrewarded dimension (IED extradimensional shift errors; Stage 8), which is analogous to the set shifting dimension of the Wisconsin Card Sorting Test and related to functions of the dorsolateral prefrontal cortex [96], [100]. The fairly isolated reversal learning impairment in HIV+ women represents a reduced response to negative feedback [101], manifesting as a decreased ability to apprehend signals suggesting that environmental contingencies have changed.

Further, HIV-related impairments in decision-making abilities were isolated to deficits in the quality of their decision-making (CGT), such that HIV+ women more often chose the option that was less likely to be correct, reflective of poorer probabilistic judgment. We did not find group differences in proportion of points placed at risk across different odds ratio trials (CGT – risk adjustment). Both groups equivalently risked a higher proportion of their points when the odds were more in their favor. The lack of greater “risky decision-making” in HIV+ women diverges from prior reports of riskier decisions on the Iowa Gambling Task (IGT) in mixed-gender [26] and male-only HIV+ samples [25]. Along with possible gender differences, it is plausible that task-specific demands explain this discrepancy. The CGT captures simpler core cognitive processes as compared to more complex processes assessed by the IGT, such that the CGT provides more tangible and pertinent risk-exposure information on each gambling trial and minimizes the inter-trial learning component.

Overall, the rather limited array of HIV-related impairments detected in this study appears to diverge from the existing literature. Two characteristics of the current study may provide context for this apparent discrepancy. First, the narrower array of deficits we observed on conventional tests may reflect our comparator group approach, as we chose not to rely on normative data. In contrast, approximately 70% of all studies published in the last 5 years have utilized published normative data to characterize cognitive functioning in HIV+ samples (results available upon request). These studies have generally found greater and more widespread impairment patterns. Indeed, when neurocognitive performance of the current HIV+ sample was indexed to normative data, HIV-related impairment appeared magnified and included impairments in learning, memory and working memory as well as deficits in fine motor speed and dexterity. These normative-based findings, however, fail to account for important comorbidities that may negatively modify neurocognition (see below). By using a matched comparator sample approach, we estimated impairments that are apt to be more exclusively related to HIV+ viral status and/or its treatment.

Secondly, the more circumscribed pattern of impairments may reflect differences in the manifestations of HIV-related neurocognitive deficits in a female sample. The few prior studies that have examined women only also detected relatively limited impairments on conventional tests, which often were restricted to psychomotor and processing speed deficits [33], [51], [84]. Consequently, findings from men-only and mixed samples may not directly relate to women-only samples [102]. Ultimately, establishing normative data *relevant* to specific HIV+ sub-populations appears to be crucial, as suggested by others [31], [33].

The current research also provided further insights into the differential impact of various potential modifiers of neurocognitive functioning, including HIV-related factors and secondary comorbidity. Notably, we determined that suffering from depression and active HCV infection were related to worse neurocognition and accounted for a substantial portion of overall neurocognition (i.e., CCI) observed in the HIV+ group. Further, the presence of detectable HIV viral load predicted additional neurocognitive attenuation and accounted for significant performance variability beyond comorbidity. This suggests that inherently occurring confounds of HIV infection account for some, but not all, of the neurocognitive variation. Interestingly, these same comorbidities did not account for significant neurocognitive performance variability in HIV− individuals.

To our knowledge our study is only the second (after Malan- Müller et al. 2013) [56], to examine the relationship between LTL and neurocognition in HIV+ women. Shorter LTL has been associated with HIV infection [55]–[57] and linked to worse neurocognitive function in both normal aging [67]–[69] and non-HIV related dementia [64]–[66]. Nonetheless, we found no difference in the LTLs of the HIV+ versus HIV− groups in this sample. Further, in contrast to Malan-Müller et al. (2013) [56], we did not identify significant associations between neurocognition (both overall and on select tests) and LTL in the HIV+ participants. Notably, the present sample was better educated and had much higher rates of cART than the sample investigated by Malan-Müller and colleagues.

Interestingly, shorter LTL was associated with poorer overall cognition in HIV− participants, after accounting for variance explained by demographic variables and lifetime substance use. Perhaps LTL is not a uniform marker of cellular aging in select populations or when under certain influences [103]. Indeed, it is plausible that in addition to HIV status, our groups differed on other unidentified factors that alter TL, such as father's age at birth [57], amount of physical activity [104], or estrogen levels [105]. These factors may be differentially associated with the groups; thus, obscuring the associations between LTL and neurocognition.

Like all research, several limitations should be noted. The current study was not designed to longitudinally track the effects of cART on neurocognition. This line of inquiry may be important as certain medications have greater brain penetration and potentially greater protective and beneficial effects [106]. Future longitudinal studies may also clarify the unexpected finding that select aspects of neurocognition were related to a longer history of HIV and ART treatment, a finding that has also been previously reported [42]. Individuals who are more adherent to longer-term treatment may show moderation of their neurocognitive impairment. Alternatively, sample ascertainment factors may be operating, such that a subset of women in the sample with higher cognitive functioning who received regular, long-term, services within the clinic may have been disproportionately represented.

The self-report nature of substance use in the entire sample could have been improved through urine drug toxicology screens. Like most past studies we did not include standardized protocols addressing substance abuse and dependence; however, we did capture a history of past and recent use. Another limitation was in using self-report in the identification of HCV status in the control group, with subsequent qPCR testing of the identified control participants. Self-report of HCV status in controls may represent a confound in the group comparisons. This is unlikely, however, in that several control participants would have to be unaware of their HCV infection, given the slightly lower, yet non-significant, difference in the percentage of control participants reporting HCV (24.4%) relative to the percentage of participants in the HIV+ group with HCV (34.6%).

It is also possible that certain characteristics of our HIV+ sample may account for the findings we observed, including the limited neurocognitive deficits detected. These characteristics include exclusively enrolling Canadian women being treated in a specialty clinic for HIV (note that the HIV+ women were fairly representative of the Canadian HIV+ population), the fact that the majority of HIV+ women were currently virally suppressed, and exclusion of women with moderate to severe head injuries. These characteristics may influence the generalizability of our study to other research contexts.

In conclusion, we found circumscribed neurocognitive impairments in a sample of HIV+ women that was well-matched to a comparison group, namely slower information processing, poorer reversal learning, and suppressed quality of decision-making. The combined deficiencies in quality of decision-making and reversal learning may further indicate impaired behavioural adaptation [107], which involves incorporating relevant information from the environment to modify behaviour in a more beneficial manner. The neurocognitive impairments detected in this study could have important implications for day-to-day functioning. The slowed information processing reflects a reduction in the speed with which new information can be processed and may reflect longer times to complete common everyday tasks [108], [109], as well as possibly affecting how well patients process new information during medical appointments. This deficit, in combination with reduced reversal learning, may make completing instrumental activities of daily living more cumbersome [92], [97], such as learning and maintaining new medication regimens [46], [110]. Continued research into the mechanisms that maintain HIV-relevant neurocognitive dysfunction in the cART era, along with the underlying brain circuitry, may provide targets for interventions that could have meaningful consequences in the real world. Additionally, this research continues to highlight the importance of evaluating depression and HCV in HIV+ women, suggesting that implementation of medication for HCV and/or treatment for depression may mitigate neurocognitive impairment, and consequently further reduce deficits observed in day-to-day activities (e.g., work, driving ability, etc.).
